# AST1306, A Novel Irreversible Inhibitor of the Epidermal Growth Factor Receptor 1 and 2, Exhibits Antitumor Activity Both In Vitro and In Vivo

**DOI:** 10.1371/journal.pone.0021487

**Published:** 2011-07-18

**Authors:** Hua Xie, Liping Lin, Linjiang Tong, Yong Jiang, Mingyue Zheng, Zhuo Chen, Xiaoyan Jiang, Xiaowei Zhang, Xiaowei Ren, Wenchao Qu, Yang Yang, Hua Wan, Yi Chen, Jianping Zuo, Hualiang Jiang, Meiyu Geng, Jian Ding

**Affiliations:** 1 State Key Laboratory of Drug Research, Division of Antitumor Pharmacology, Shanghai Institute of Materia Medica, Chinese Academy of Sciences, Shanghai, China; 2 Shanghai Allist Pharmaceuticals, Shanghai, China; 3 State Key Laboratory of Drug Research, Drug Discovery and Design Center, Shanghai Institute of Materia Medica, Chinese Academy of Sciences, Shanghai, China; 4 Laboratory of Immunopharmacology, State Key Laboratory of Drug Research, Shanghai Institute of Materia Medica, Chinese Academy of Sciences, Shanghai, China; 5 Shuguang Hospital affiliated with Shanghai University of Traditional Chinese Medicine, Shanghai, China; Florida International University, United States of America

## Abstract

Despite the initial response to the reversible, ATP-competitive quinazoline inhibitors that target ErbB-family, such a subset of cancer patients almost invariably develop resistance. Recent studies have provided compelling evidence that irreversible ErbB inhibitors have the potential to override this resistance. Here, we found that AST1306, a novel anilino-quinazoline compound, inhibited the enzymatic activities of wild-type epidermal growth factor receptor (EGFR) and ErbB2 as well as EGFR resistant mutant in both cell-free and cell-based systems. Importantly, AST1306 functions as an irreversible inhibitor, most likely through covalent interaction with Cys797 and Cys805 in the catalytic domains of EGFR and ErbB2, respectively. Further studies showed that AST1306 inactivated pathways downstream of these receptors and thereby inhibited the proliferation of a panel of cancer cell lines. Although the activities of EGFR and ErbB2 were similarly sensitive to AST1306, ErbB2-overexpressing cell lines consistently exhibited more sensitivity to AST1306 antiproliferative effects. Consistent with this, knockdown of ErbB2, but not EGFR, decreased the sensitivity of SK-OV-3 cells to AST1306. In vivo, AST1306 potently suppressed tumor growth in ErbB2-overexpressing adenocarcinoma xenograft and FVB-2/N^neu^ transgenic breast cancer mouse models, but weakly inhibited the growth of EGFR-overexpressing tumor xenografts. Tumor growth inhibition induced by a single dose of AST1306 in the SK-OV-3 xenograft model was accompanied by a rapid (within 2 h) and sustained (≥24 h) inhibition of both EGFR and ErbB2, consistent with an irreversible inhibition mechanism. Taken together, these results establish AST1306 as a selective, irreversible ErbB2 and EGFR inhibitor whose growth-inhibitory effects are more potent in ErbB2-overexpressing cells.

## Introduction

The ErbB tyrosine kinase superfamily, comprising the epidermal growth factor receptor (EGFR; also known as ErbB1/HER1), ErbB2 (HER2/neu), ErbB3 (HER3) and ErbB4 (HER4), plays important roles in cancer development and progression [1]. Upon binding their cognate ligands (e.g., EGF, transforming growth factor-α), these receptors form active homodimers and heterodimers. No ligand has been identified for ErbB2; instead, this protein functions as a coreceptor by binding to other receptors in the family [2]. Activation of ErbB family receptors results in subsequent recruitment and phosphorylation of several intracellular substrates, including components of the Ras-Raf-MAPK (mitogen-activated protein kinase) and the PI3K (phosphoinositide 3-kinase)-AKT pathways, leading to mitogenic signaling and other cellular activities [3], [4]. Dysregulation of ErbB receptor activity through overexpression or mutation is associated with a number of different cancers; thus, members of the ErbB family have become important therapeutic targets in several types of cancer.

Numerous reversible ErbB tyrosine kinase inhibitors (TKIs) are currently in development for cancer therapy. Several such inhibitors have been approved for use in cancer patients, including gefinitib (ZD-1839, Iressa), erlotinib (OSI-774, Tarceva) and lapatinib (GW572016, Tykerb) [5], [6], [7]. However, treatment with these reversible TKIs produces objective responses in a rather small subset of patients, possibly corresponding to individuals with activating mutations in the EGFR tyrosine kinase domain, such as the L858R mutation [8], [9]. Despite positive initial response, these patients almost invariably develop acquired secondary resistance, such as substitution of threonine 790 with methionine (T790M), to these reversible inhibitors and relapse after several months [10], [11], [12], which accounts for about half of all cases of resistance to gefitinib and erlotinib [13], [14]. Therefore, resistance to reversible ErbB inhibitors has emerged as a significant clinical problem.

Recent studies have revealed that acquired EGFR mutations remain sensitive to irreversible ErbB inhibitors. Oral administration of these irreversible inhibitors produces significant anti-tumor activity in a variety of human tumor xenograft models that express or overexpress ErbB family members, especially those that contain the EGFR double mutation, L858R/T790M [15], [16]. Thus, irreversible EGFR inhibitors potentially provide a second-line treatment for managing resistance to reversible EGFR inhibitors. Several of these inhibitors, namely HKI-272 [17], EKB-569 [18], BIBW2992 [19], [20] and PF00299804 [21], are currently undergoing clinical testing; however, none of them have yet received approval by FDA.

We rationally designed and synthesized a series of quinazoline derivatives based on the chemical structure of lapatinib, combining the key chemical group of irreversible EGFR inhibitors. One such derivative, AST1306, stood out in these screens and was selected for further evaluation. In this report, we evaluated the *in vitro* and *in vivo* antitumor activity of AST1306 and identified it as a novel irreversible ErbB family inhibitor. AST1306 potently inhibits wild-type EGFR and ErbB2, as well as EGFR mutants, in both cell-free and intact cell assays. Moreover, ErbB2-overexpressing tumors are more sensitive to the growth-inhibitory effects of AST1306 than are EGFR-overexpressing tumors, both *in vitro* and *in vivo*. Our data establish AST1306, which is currently in phase I clinical trial in China, as a novel irreversible ErbB inhibitor that deserves further development.

## Materials and Methods

### Cell culture

SK-OV-3, BT474, MDA-MB-468, Calu-3, A-549, NCI-H23, NCI-H1975 and NIH3T3 cells were obtained from the American Type Culture Collection (Rockville, MD). HO-8910 cells were obtained from the cell bank of the Chinese Academy of Sciences. MCF-7 cells were obtained from the Japanese Foundation of Cancer Research. NIH3T3 cells that overexpressed EGFR T790M/L858R mutant (NIH3T3-EGFR T790M/L858R) were constructed and maintained in our laboratory. All cell lines were maintained in strict accordance with the supplier's instructions and established procedures.

### Cell proliferation, tyrosine kinase, and Western blot assay

The cell proliferation assay was evaluated using SRB (Sulforhodamine B) assay treated for 72 h with indicated concentrations of AST1306. The tyrosine kinase activities were determined using enzyme-linked immunosorbant assay (ELISA). The serine/threonine kinase activities were detected by Caliper Mobility Shift Assay on EZ Reader (Caliper Life Sciences, MA) according to the instruction. For analysis of receptor tyrosine kinase phosphorylation and downstream signal transduction pathways in human cancer cells, starved cells were treated with AST1306 for 4 h and stimulated with EGF (50 ng/ml) for 15 min, and then collected and processed for Western blot analysis (ref. [22], [23], [24]; see Text S1 for a complete description).

### Molecular docking simulation

The crystal structure of EGFR kinase domain in complex with lapatinib (PDB entry 1XKK) [25] was used as EGFR target for molecular docking simulation. 3D models of the ErbB2 kinase domain was created according to highly homologous protein EGFR by employing MODELLER program [26]. Ten models of each enzyme were generated and all these 3D structures were optimized with conjugate gradient minimization scheme followed by a restrained simulated annealing molecular dynamics simulation. The one with the lowest value of the objective function was selected as a representative target model for further study.

Structure-based analysis was performed using the docking program AUTODOCK4.0 [27]. The Lamarckian genetic algorithm (LGA) was applied with the step size set to 0.2 Å for translation and 5° for orientation and torsion. The number of generation, energy evaluation, and docking runs were set to 500,000, 2,500,000 and 20, respectively. Ligand poses with the lowest binding energy were then used to analyze the interaction mode between the ligand and its different targets.

### Irreversibility assessments for AST1306

Rapid dilution experiments as described previously [25] were used to demonstrate irreversible binding of AST1306 to EGFR and ErbB2. Nine microliters of 100-fold normal amount of enzyme (800 nM for EGFR, 1800 nM for ErbB2) was mixed with 1 µL of AST1306 at a final concentration of 100-fold IC_50_ for each enzyme or with vehicle control (DMSO). Lapatinib was used as a positive control compound. After incubation at room temperature for 30 minutes, 1 µL of the mix solution of was diluted into 99 µL of a solution containing substrate peptide (sequence: 5-FAM-EEPLYWSFPAKKK-CONH_2_, 1.5 µM) and ATP (2.3 µM for EGFR, 15 µM for ErbB2). The microplate was placed in the EZ Reader (Caliper Life Sciences, MA) and wells were repeatedly sampled for 180 minutes.

### Small interfering RNA transfection

Small interfering RNA (siRNA) against EGFR or ErbB2, as well as control siRNA, were designed and synthesized by Shanghai GenePharma Co. Ltd. One pair of ErbB2 siRNA (sequence: 5′ CGUUUGAGUCCAUGCCCAATT 3′), and two pairs of EGFR siRNA (#1 sequence: 5′ GGUAGUAAAUAUGAAACUATT 3′; #2 sequence: 5′ GUCCUUGGGAAUUUGGAAATT 3′) were applied in this study. Different kind of siRNA (final concentration: 50 nmol/L) was transfected to SK-OV-3 cells with oligofectamine reagent (Invitrogen) according to the manufacturer's instruction. AST1306 treatment was done 48 h after transfection. Growth-inhibitory effects of AST1306 on the cells were determined by SRB assay.

### Suppression of tumor growth in human tumor xenograft models and tumor extract analyses

The mice were housed and maintained under specific-pathogen free (SPF) conditions. All animal procedures were performed according to the protocol approved by the Institutional Animal Care and Use Committee at Shanghai Institute of Materia Medica, Chinese Academy of Sciences (SIMM-AE-DJ-2010-01). Under sterile conditions, well-developed tumors were cut into 1-mm^3^ fragments and transplanted s.c. into the right flank of nude mice using a trocar. When the tumor reached a volume reached 100 to 200 mm^3^, the mice were randomly assigned into control and treatment groups. Control groups were given vehicle alone, and treatment groups received AST1306 (100, 50 and 25 mg/kg) or lapatinib (50 mg/kg) (p.o, twice daily). The sizes of the tumors were measured twice per week using microcalipers. The tumor volume (V) was calculated as follows: V = (length×width^2^)/2. The body weights of the mice were observed simultaneously.

To assess the pharmacodynamic effect in tumors, mice bearing established (∼200 mm^3^) SK-OV-3 xenograft tumors were treated with a single dose of AST1306 at 100 mg/kg. Tumors were harvested at indicated times (n = 3 per time point) after treatment, immediately frozen in liquid nitrogen, and then homogenized in 500 µL protein extraction solutions (SBS Genetech, Shanghai, China). Tumor extracts were subjected to SDS-PAGE followed by immunoblotting.

### Antitumor activity against spontaneity breast cancer in FVB-2/N^neu^ transgenic mouse

Thirty eight- to forty four-week-old female FVB/N^neu^ mice were obtained from Shanghai Laboratory Animal Center. The animals were housed under specific pathogen-free conditions. The solution of AST1306 was suspended in 0.5% hydroxypropyl methylcellulose (HPMC), grinding with agate mortar. Mice were administered AST1306 at dosage of 100, 50 and 25 mg/kg twice daily and treated with lapatinib (50 mg/kg) as comparison. Tumors were measured twice a week in two dimensions, using a caliper, and the tumor volume was calculated according to the formula L×W×W/2.

### Statistical analysis

Data are expressed as the mean ± SD, and differences were considered significant at *P*<0.05 determined by *t* test.

## Results

### AST1306 selectively inhibits the tyrosine kinase activities of EGFR and ErbB2 in vitro

**Figure 1:**
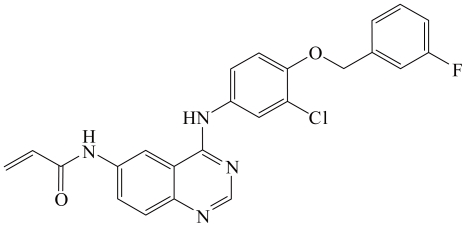
Chemical structure of AST1306.

The compound AST1306 was synthesized as described in Text S1, and its chemical structure is shown in Fig. 1. We first examined the inhibitory activities of AST1306 by performing *in vitro* assays against EGFR, ErbB2 and 23 other kinases. AST1306 was a potent inhibitor of EGFR and ErbB2 in a cell-free assay, exhibiting IC_50_ values of 0.5 and 3 nmol/L, respectively (Table 1). Notably, AST1306 was more than 3000-fold selective for ErbB family kinases over other kinase families tested in the present study (Table 1). AST1306 is thus a potent selective inhibitor of EGFR and ErbB2.

**Table 1:** In vitro inhibitory activity of AST1306 on different kinds of kinase.

Kinase	IC_50_ a, nmol/L
EGFR	0.5±0.2
ErbB2	3.0±1.5
ErbB4	0.8±0.3
Flt-1	>10000
KDR	>10000
PDGFRα	>10000
PDGFRβ	>10000
c-Src	>10000
c-Met	>10000
c-Kit	>10000
IGF1R	>10000
FGFR1	>10000
RON	>10000
Abl	>10000
EphA2	>10000
EphB2	>10000
Tie2	>10000
SYK	>10000
PKCb2	>10000
p70S6K	>10000
JAK2	>10000
Pim2	>10000
AKT1	>10000
AUR A	>10000
CDK2	>10000
GSK3b	>10000
EGFR T790M/L858R	12±2

a IC_50_, the concentration of AST1306 inducing 50% inhibition of kinase activity. IC_50_s were calculated by Logit method and expressed as means ± SD.

### AST1306 irreversibly binds EGFR and ErbB2

**Figure 2:**
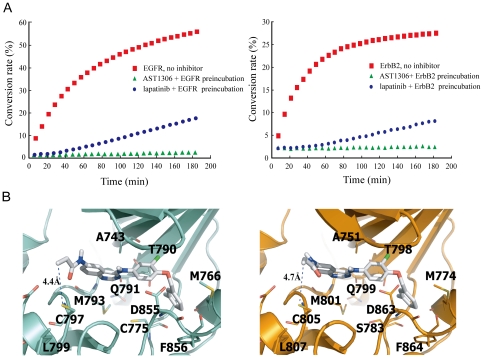
AST1306 irreversibly binds EGFR and ErbB2. A, Recombinant EGFR (left panel) or ErbB2 (right panel) was preincubated for 30 min with or without AST1306 or lapatinib at a concentration 100-fold in excess of the respective IC_50_ value in the absence of substrate, and then diluted and assayed for enzymatic activity. B, The binding mode of AST1306 with EGFR (left panel) or ErbB2 (right panel) predicted by the molecular docking simulations. Key distances and related amino acid residues are shown in the figure and discussed in detail in the text.

AST1306 was designed to be an irreversible ErbB family inhibitor. To verify this, we determined the reversibility of EGFR and ErbB2 binding using a dilution method. In this approach, an amount of receptor 100 times that normally used in a reaction is preincubated for 30 min with a concentration of AST1306 100-fold greater than the IC_50_ value or with the vehicle control. After incubating, the enzyme/inhibitor mixture is diluted 100-fold (i.e., to normal reaction conditions), reaction buffer plus ATP and substrate are added, and receptor kinase activity is measured continuously. In general, a reversible inhibitor dissociates quickly, allowing immediate recovery of enzymatic activity, whereas a slowly reversible inhibitor allows a gradual increase in activity. In contrast, an irreversible inhibitor prevents recovery of enzymatic activity. As shown in Fig. 2A, virtually no EGFR or ErbB2 activity was recovered after incubating with AST1306, convincingly demonstrating that AST1306 is an irreversible inhibitor of EGFR and ErbB2. Reactions containing lapatinib, a slowly reversible inhibitor of EGFR and ErbB2, used as a control, revealed a slow recovery of the enzymatic activity, consistent with previous report [25].

### AST1306 is predicted to covalently bind to critical amino acids of EGFR and ErbB2

The observation that AST1306 potently, selectively and irreversibly inhibited EGFR and ErbB2 prompted us to further investigate the mode of AST1306 binding to its targets. Using a molecular docking method and a previously described molecular model of EGFR with lapatinib, we docked AST1306 into the ATP-binding pocket of the EGFR kinase domain (Fig. 2B, left panel). The results of this analysis showed that AST1306 was surrounded by hydrophobic residues in the ATP-binding pocket of EGFR. Importantly, the β-carbon atom of the Michael acceptor is predicted to be located at a distance of 4.4 Å from the sulfur atom of Cys-797, which is known to be a critical residue for irreversible inhibitor binding and should be easily accessible for the covalent interaction. A similar mode was also predicted for AST1306 binding to our homology model of ErbB2. As shown in Fig. 2B (right panel), in addition to the hydrophobic interactions with ErbB2, AST1306 was predicted to form covalent interaction with Cys-805, which is also known to be a critical residue for irreversible inhibitor binding to ErbB2. These results demonstrate that AST1306 might covalently bind to specific amino acid residues of EGFR and ErbB2, consistent with previous reports of irreversible inhibitors, such as EKB-569 [28].

### AST1306 inhibits the tyrosine kinase activity of the EGFR mutant T790M/L858R in cell-free and intact cell assays

**Figure 3:**
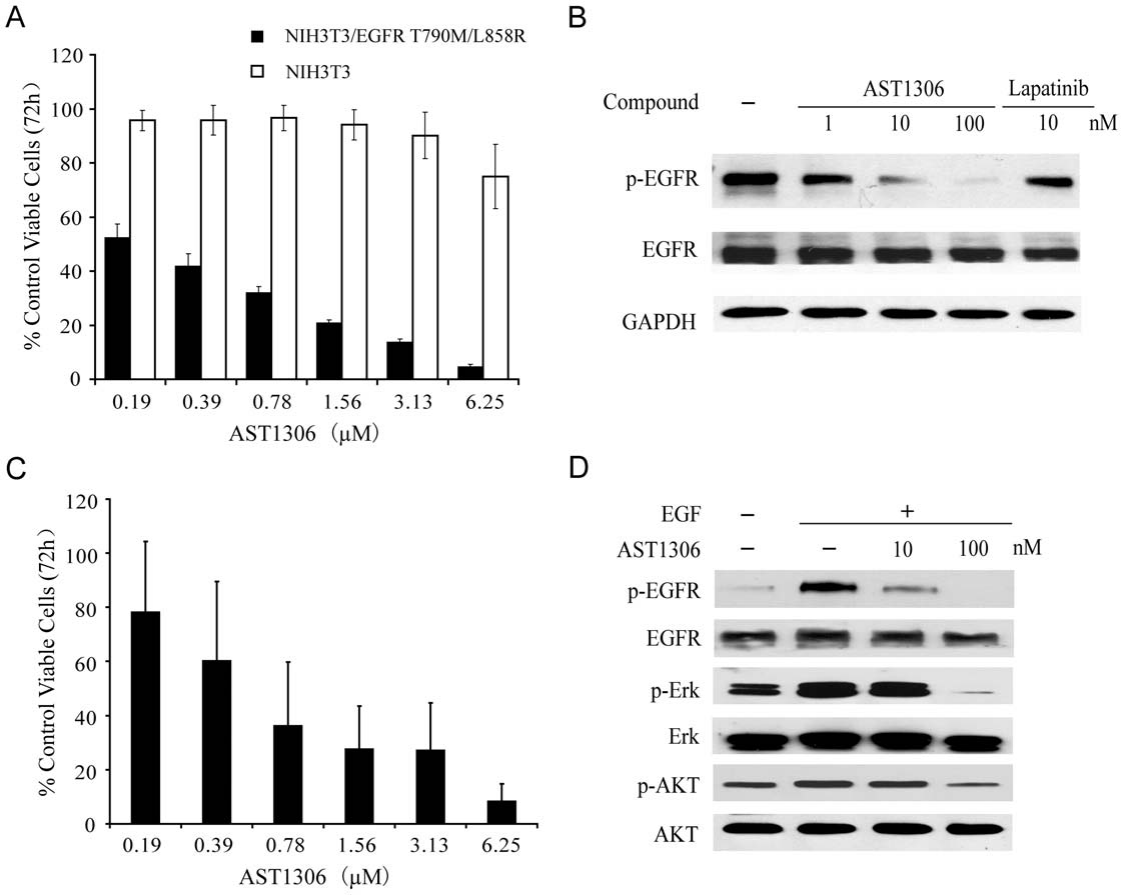
Effects of AST1306 on cells harboring the EGFR T790M/L858R double mutant. A, Inhibition by AST1306 of the proliferation of NIH3T3 parental cells and NIH3T3 cells engineered to express EGFR T790M/L858R double mutant. Cells were treated with the indicated concentrations of AST1306 for 72 h. Cell viability was determined using sulforhodamine B assay. IC_50_ values were determined based on the results of three independent experiments. B, AST1306 inhibits the phosphorylation of EGFR in NIH3T3-EGFR T790M/L858R cells. Cells were cultured in the presence of different concentrations of AST1306 for 6 h and harvested. Whole-cell lysates were assayed for total EGFR and phosphorylation of EGFR by immunoblotting. A representative anti-GAPDH immunoblot is shown as a loading control. C, Growth inhibition of NCI-H1975 cells by AST1306. Cell viability was determined using sulforhodamine B assay. D, AST1306 inhibits EGF-induced phosphorylation of EGFR and that of downstream signaling molecules in NCI-H1975 cells.

The EGFR T790M/L858R mutant confers resistance in patients treated with reversible ErbB inhibitors, but is known to retain sensitivity to irreversible ErbB inhibitors. Accordingly, we tested whether AST1306 possesses inhibitory activity toward EGFR T790M/L858R in a cell-free system. AST1306 potently inhibited the EGFR T790M/L858R double mutant, exhibiting an IC_50_ value of 12±2 nmol/L (Table 1), which was approximately 500-fold more potent than lapatinib (IC_50_>5000 nmol/L; data not shown). We next determined the inhibitory activity of AST1306 toward this EGFR double mutant at the cellular level. As shown in Fig. 3A, AST1306 induced a significant, concentration-dependent inhibition of the growth of HIH3T3-EGFR T790M/L858R cells. In contrast, it was much less potent toward parental NIH3T3 cells. In addition, AST1306 effectively inhibited EGFR phosphorylation in HIH3T3-EGFR T790M/L858R cells, whereas lapatinib showed much weaker inhibitory effect on it. (Fig. 3B). Moreover, AST1306 inhibited the growth of NCI-H1975 cells that harbor the EGFR T790M/L858R mutation in a concentration-dependent manner (Fig. 3C), and blocked phosphorylation of EGFR and downstream pathways as well (Fig. 3D). These results suggest that AST1306, like other irreversible inhibitors, may overcome the acquired resistance to reversible inhibitors.

### AST1306 inhibits the phosphorylation of EGFR and ErbB2, and downstream signaling in human cancer cells

**Figure 4:**
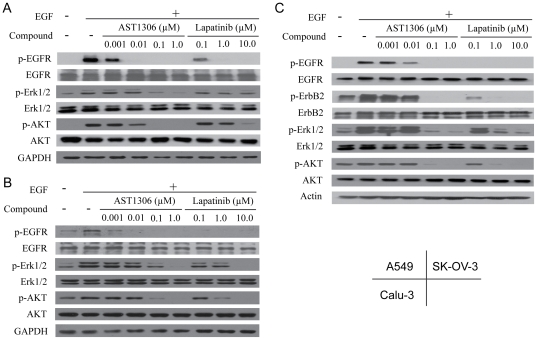
AST1306 inhibits the activation of tyrosine kinases and downstream signaling pathways in A549 cells (A), Calu-3 cells (B) and SK-OV-3 cells (C). Cells were starved in serum-free medium for 24 h and then cultured in the presence of varying doses of AST1306 for 4 h at 37°C in medium without FBS. EGF (50 ng/ml) was then added. After a 15-min incubation at 37°C, whole -cell lysates were harvested and assayed by Western blotting.

We next evaluated the targets inhibition of AST1306 in human cancer cells that overexpress EGFR and/or ErbB2. AST1306 dose-dependently and markedly inhibited EGF-induced EGFR phosphorylation in A549 cells, which are known to express high levels of EGFR. As shown in Fig. 4A, the bands of phospho-EGFR almost disappeared after the treatment of AST1306 at 0.01 µM concentration. Activity of the downstream pathways of EGFR was also decreased in dose-dependent manner after treatment with AT1306, as evidenced by decreased phosphorylation of Erk1/2 and AKT (Fig. 4A). Of note, the bands of phospho-Erk1/2 and phospho-AKT became undetectable at much higher concentration of AST1306 than that of phospho-EGFR inhibition, which might due to the crosstalk between different signaling pathways or other potential mechanism of this compound.

Moreover, we also detected the targets inhibition and downstream signaling inhibition of AST1306 in other cancer cell lines. Similar results were also obtained in ErbB2-overexpressing Calu-3 cells (Fig. 4B) and SK-OV-3 cells that overexpress both EGFR and ErbB2 (Fig. 4C). Notably, AST1306 was a >10-fold more potent inhibitor of targets and the downstream signaling than the reference compound lapatinib in the human cancer cells tested.

### AST1306 inhibits the proliferation of human cancer cells, with ErbB2- overexpressing cells exhibiting more sensitivity

**Figure 5:**
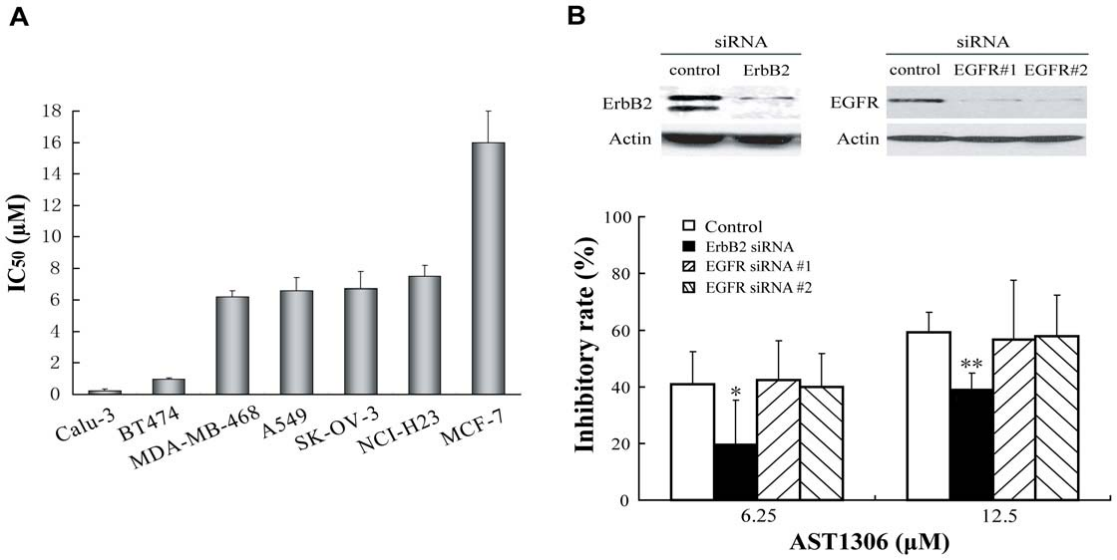
Antiproliferative activities of AST1306 in vitro. A, Antiproliferative activities of AST1306 in human cancer cell lines *in vitro* were determined using a sulforhodamine B assay. B, Knockdown of ErbB2, but not EGFR, leads to AST1306 resistance in SK-OV-3 cells (*P<0.05; **P<0.01). Each experiment was repeated three times independently.

The antiproliferative effects of AST1306 were then evaluated in a panel of human cancer cell lines with varying levels of EGFR and ErbB2 expression. As shown in Fig. 5A, AST1306 effectively suppressed the proliferation of human cancer cell lines; however, the IC_50_ values varied widely among them. The Calu-3 lung adenocarcinoma and BT474 breast cancer cell line, containing high levels of ErbB2, were more sensitive to AST1306, with IC_50_ values of 0.23 and 0.97 µmol/L, respectively. In contrast, cell lines with high levels of EGFR but lower levels of ErbB2 (MDA-MB-468, A549 and NCI-H23) or high levels of both ErbB2 and EGFR (SK-OV-3) were less sensitive to AST1306, with an IC_50_ values ranging from 6.2 to 7.5 µmol/L. The MCF-7 cell line, which expresses low levels of both EGFR and ErbB2, was the least sensitive to AST1306, with IC_50_ value of 16.0 µmol/L. Moreover, we also detected the activity of AST1306 on anchorage-independent growth of SK-OV-3 cells and A549 cells, respectively. As shown in Figure S1, AST1306 can dramatically inhibit the growth of both tumor cells on soft agar, and SK-OV-3 cells exhibited much more sensitivity than that of A549 cells. These results indicate that AST1306 inhibits the proliferation of human cancer cell lines *in vitro*, and suggest that ErbB2 expression is associated with a consistently higher sensitivity to AST1306 across the various cell lines tested.

### Silencing of ErbB2, but not EGFR, decreases the sensitivity of SK-OV-3 cells to AST1306

The above results revealed that although AST1306 potently inhibited EGFR and ErbB2 enzymatic activities in a cell-free assay and significantly dephosphorylated EGFR and ErbB2 at the cellular level, proliferation was more sensitive to inhibition by AST1306 in ErbB2-overexpressing cell lines than in EGFR-overexpressing cell lines. To address this discrepancy, we examined AST1306 sensitivity upon knockdown of EGFR and ErbB2. First of all, we tested the activity of siRNA transfection on proliferation of SK-OV-3 cells, and found that it had no obvious effects on the cell proliferation (Figure S2). And then, the siRNA was transfected into SK-OV-3 cells and treated with AST1306 or vehicle. As shown in Figure 5B, SK-OV-3 cells transfected with ErbB2 siRNA were significantly more resistant to treatment with AST1306 at concentrations of 6.25 µmol/L (*p*<0.05) or 12.5 µmol/L (*p*<0.01). However, knocking down EGFR expression using two independent pairs of EGFR siRNAs did not significantly affect the antiproliferative activity of AST1306 (Fig. 5B). These results suggest that ErbB2 plays a more important role in the sensitivity to the antiproliferative effects of AST1306 than does the EGFR, at least in SK-OV-3 cells.

### AST1306 potently inhibits tumor growth in ErbB2-overexpressing xenograft models

**Figure 6:**
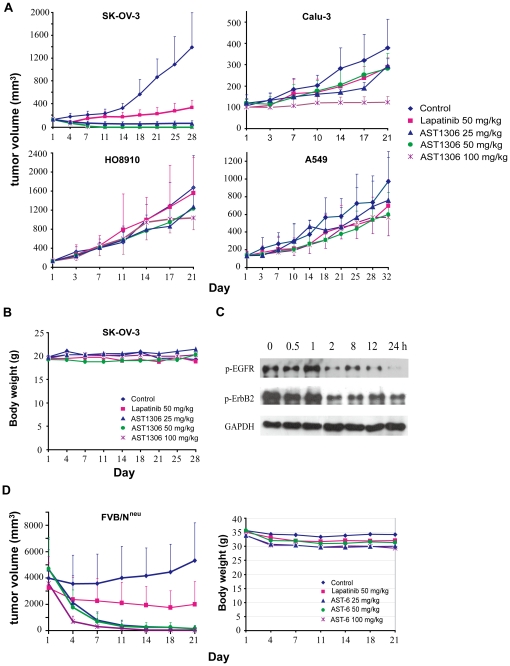
In vivo activity of AST1306 in xenograft models and FVB-2/N^neu^ transgenic mouse model. A, Inhibitory effects of AST1306 on tumor growth in human tumor xenograft models. Body weights of the nude mice were measured concurrently (B). C, Inhibition of target (EGFR and ErbB2) activation by AST1306 in tumors. Nude mice bearing established (∼200 mm^3^) SK-OV-3 tumors were treated with a single dose of AST1306 (100 mg/kg). Tumors were harvested at the indicated times after treatment and immediately frozen in liquid nitrogen. Western blot analysis was performed to determine the levels of phosphorylated EGFR and ErbB2. D, Activity of AST1306 in FVB-2/N^neu^ transgenic mouse model.

Given its encouraging *in vitro* activity, we examined the antitumor efficacy of AST1306 *in vivo*. Specifically, we measured the antitumor activity of AST1306 in two different human ovarian cancer xenograft nude mice models (SK-OV-3 and HO-8910), and two different human lung cancer xenograft nude mice models (Calu-3 and A549). SK-OV-3 and Calu-3 are known to be ErbB2-overexpressing tumors, whereas HO-8910 and A549 tumors express low levels of ErbB2. As shown in Fig. 6A, twice daily oral administration of AST1306 caused a dramatic suppression of tumor growth in SK-OV-3 and Calu-3 xenograft models. In SK-OV-3 models, tumors almost completely disappeared after treatment with AST1306 for 7 d. In contrast, AST1306 only slightly suppressed tumor growth in HO-8910 and A549 xenograft models (Fig. 6A). These results demonstrate that the antitumor efficacy of AST1306 is greater in ErbB2-overexpressing tumor models than in models expressing low levels of ErbB2, consistent with the results of *in vitro* proliferation experiments and siRNA-mediated inhibition assays. Body weights of nude mice measured concurrently showed no obvious differences during the treatment (Fig. 6B), demonstrating that AST1306 was well tolerate. Lapatinib also exhibited antitumor activity in these ErbB2-overexpressing tumor models, but AST1306 was more efficacious than lapatinib in the SK-OV-3 xenograft tumor model when given at the same dose and schedule.

We also examined inhibition of target activity by measuring EGFR and ErbB2 phosphorylation levels in tumor extracts at indicated times after a single administration of AST1306. As shown in Fig. 6C, inhibition of EGFR and ErbB2 activation by AST1306 was clearly evident in tumors within 2 h after treatment. Notably, these inhibitory effects lasted at least 24 h.

### AST1306 inhibits the tumor growth of FVB-2/N^neu^ transgenic mouse model

To further confirm that AST1306 possesses inhibitory activities in ErbB2-overexpressing models, we evaluated the antitumor activity of AST1306 in FVB-2/N^neu^ transgenic mouse model, which expresses the ErbB2/Neu proto-oncogene and closely recapitulates the ontogeny and progression of human breast cancer [29]. As shown in Fig. 6D (left panel), oral administration of AST1306 twice daily for 3 wk caused a dramatic suppression of tumor growth in the FVB-2/N^neu^ models; after treatment for 11 d, tumors almost completely disappeared. The body weights of the mice decreased by less than 20% during treatment (Fig. 6D, right panel).

## Discussion

Resistance to reversible ErbB tyrosine kinase inhibitors has recently emerged as a crucial problem for targeted cancer therapy. The development of additional irreversible inhibitors, which have proven to be effective in overcoming treatment-resistant EGFR mutant, is warranted. In the present study, we have provided convincing evidence that AST1306 is a novel irreversible inhibitor of EGFR and ErbB2, which is superior to lapatinib. Using molecular docking models, we demonstrated that AST1306 can dock into the ATP-binding pocket of EGFR and ErbB2. Notably, the interactions between the Michael acceptor functional group of AST1306 and the corresponding critical cysteines of EGFR and ErbB2 are likely to result in covalent binding of AST1306 to the enzymes.

The most exciting potential for irreversible inhibitors is their ability to inhibit mutant forms of ErbB family members. EGFR L858R, one of the most common activating mutations, allows gefitinib to move deeper into the binding pocket, enhancing the interaction between gefitinib and the receptor [30]. T790M, a secondary resistance mutation in the EGFR kinase domain, leads to the steric hindrance in the binding of reversible EGFR inhibitors [31], and/or increases the affinity for ATP [32], and thus results in resistance to reversible inhibitors. The EGFR T790M/L858R double mutation is a common mutation that appears in clinical lung cancer patients treated with reversible inhibitors [10]. Our studies showed that AST1306 potently inhibited the EGFR T790M/L858R double mutant in both cell-free and cell-based assay, including NIH3T3 cells engineered to express the T790M/L858R mutant and NCI-H1975 cells, which endogenously express this mutant. These findings provide compelling evidence that AST1306, as an irreversible ErbB inhibitor, clearly possessed inhibitory activity toward EGFR resistant mutations, demonstrating that AST1306 may have potential utility in patients whose tumors are unresponsive to reversible inhibitors. Future investigation will be required to assess the *in vivo* efficacy of AST1306 in tumors that harbor secondary mutations in their ErbB receptor.

Mounting evidence has verified that ErbB2 is an important component of the ErbB signaling network in human cancer cells. Lacking a known endogenous ligand, ErbB2 appears to exist predominantly in an active conformation ready to heterodimerize with other ErbB family members; thus, ErbB2 containing heterodimers are more efficiently activated, especially in settings where ligand is limiting [33]. Moreover, ErbB2 expression may increase EGFR recycling to the membrane and prevent its degradation, a process that may also make a significant contribution to ErbB2 function. These properties make ErbB2 more important than other members of the ErbB family in certain respects and thus attract increasing attention in recent years. In the present study, we clearly demonstrated that high expression of ErbB2 in tumor cells were associated with enhanced sensitivity to the antiproliferative and antitumor effects of AST1306. Results from siRNA-mediated knockdown of EGFR and ErbB2 provided further support for this phenomenon. This property of AST1306 is consistent with that of lapatinib that its activity is not dependent on EGFR expression level in ErbB2-overexpressing breast cancer cells [34]. These notions provide strong evidence that, despite comparable biological inhibitory activity of AST1306 toward EGFR and ErbB2 tyrosine kinases in cell-free assays, the growth-inhibitory effect of AST1306 in human cancer cells and in xenograft tumor models tracks more closely with its anti-ErbB2 effects. Given other known irreversible ErbB inhibitors, HKI-272, BIBW2992, CI1033, and PF-00299804 are active in both EGFR- and ErbB2-dependent tumor xenograft models [15], [19], [20], [35], [36], [37], whereas EKB569 shows poorer efficacy in ErbB2-dependent tumor modes than in EGFR-dependent models [35], our results that AST1306 is more efficacious in ErbB2- than EGFR-dependent models suggest that AST1306 is somewhat different from the known irreversible ErbB family inhibitors.

Another attractive property exhibited by irreversible inhibitors is their prolonged pharmacokinetic effects. Once an irreversible inhibitor deactivates the target enzyme by covalent bond formation, its biological effects should persist even after the drug leaves the circulation. Consistent with this feature, EGFR and ErbB2 phosphorylation was inhibited for more than 24 h after AST1306 treatment in the SK-OV-3 human ovarian carcinoma xenograft model. Because the half-life of AST1306 after a single oral dose (90 mg/kg) is less than 3.6 h and 2.3 h in rats or in dogs, respectively (unpublished data), the sustained inhibition of phosphorylation seen in the xenograft studies of AST1306 allows the once- or twice-daily oral dosing. Taken together, these attributes of AST1306 would be expected to reduce the nonspecific interactions that might occur at high or prolonged plasm levels and thus reduce toxicity.

Given the critical importance of ErbB receptors in maintaining the neoplastic properties of a variety of cancers and the incompletely met medical need for effective cancer treatment, the results of the present study, combined with the better pharmacokinetic profiles and well-tolerated toxic settings (unpublished data), indicate that AST1306 deserves further study, especially in the context of human tumors that overexpress ErbB2 and/or harbor secondary mutations in their ErbB receptors.

## Supporting Information

Figure S1 **AST1306 suppressed anchorage-independent cell growth of SK-OV-3 cells (A, C) and A549 cells (B, D). Cells (8000/mL) were expose to AST1306 in 1 mL of 0.3% basal medium Eagle's agar containing 10% FBS.** The culture was maintained at 37°C in a 5% CO_2_ atmosphere for two weeks. The average colony number was calculated and colonies were photographed. *Columns*, mean of triplicate samples; *bars*, SE. Significant differences were evaluated using the Student's test (*P<0.05; ** *P*<0.01).
(TIF)

Figure S2 **Different kinds of siRNA (50 nmol/L) were transfected to SK-OV-3 cells with oligofectamine reagent (Invitrogen) according to the manufacturer's instruction.** Forty eight hours after transfection, growth effects of these siRNAs on the cells were determined by SRB assay.
(TIF)

Text S1 **Detailed description of Materials and Methods.**
(DOC)
