# Supplementary figures and images for: AST1306, A Novel Irreversible Inhibitor of the Epidermal Growth Factor Receptor 1 and 2, Exhibits Antitumor Activity Both In Vitro and In Vivo
Source: PLoS One. 2011 Jul 18;6(7):e21487. doi: 10.1371/journal.pone.0021487 (PMC3138742; doi:10.1371/journal.pone.0021487)

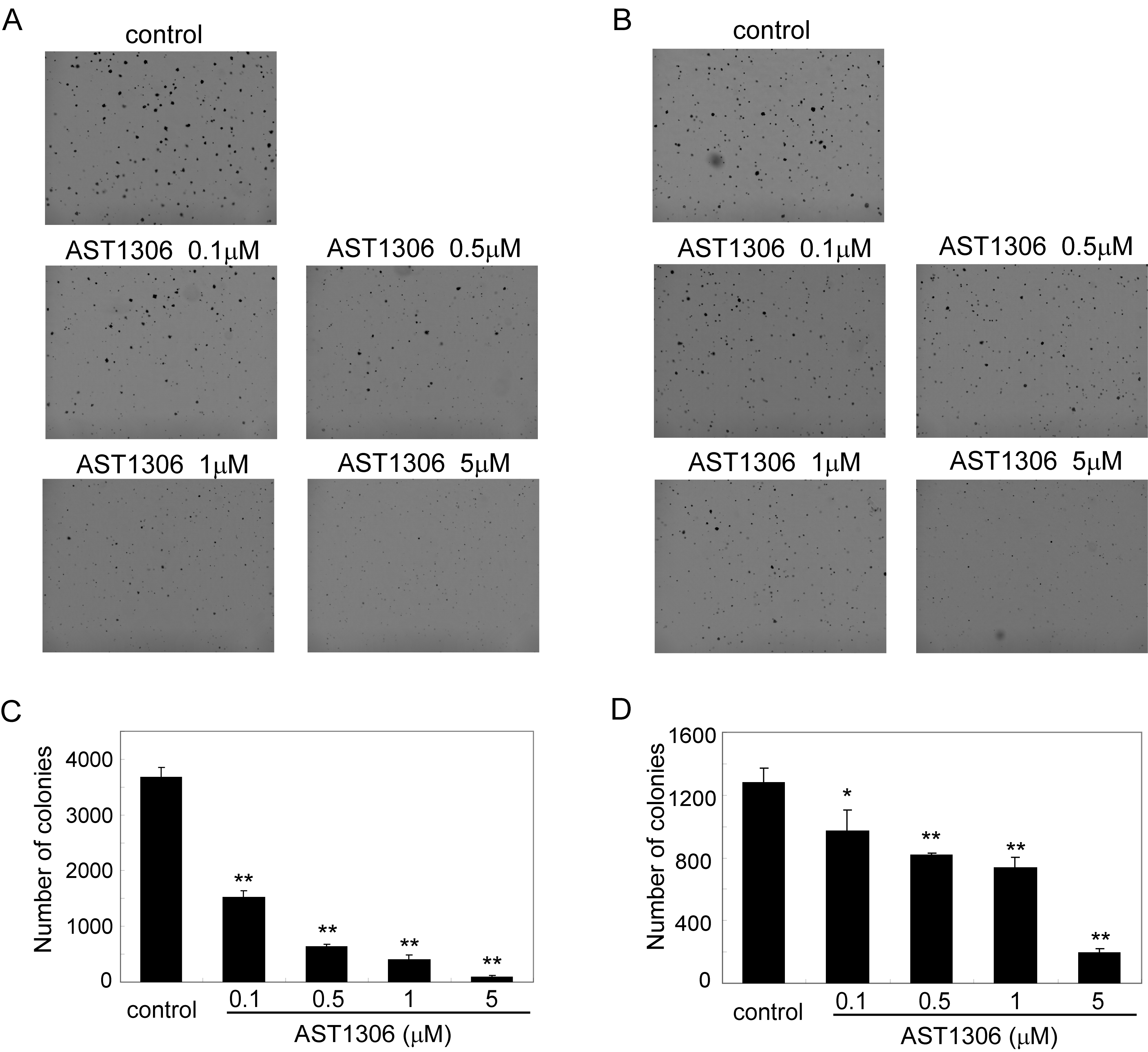

Supplement: Figure S1 — AST1306 suppressed anchorage-independent cell growth of SK-OV-3 cells (A, C) and A549 cells (B, D). Cells (8000/mL) were expose to AST1306 in 1 mL of 0.3% basal medium Eagle's agar containing 10% FBS. The culture was maintained at 37°C in a 5% CO2 atmosphere for two weeks. The average colony number was calculated and colonies were photographed. Columns, mean of triplicate samples; bars, SE. Significant differences were evaluated using the Student's test (*P<0.05; ** P<0.01). (TIF) [file pone.0021487.s001.tif]

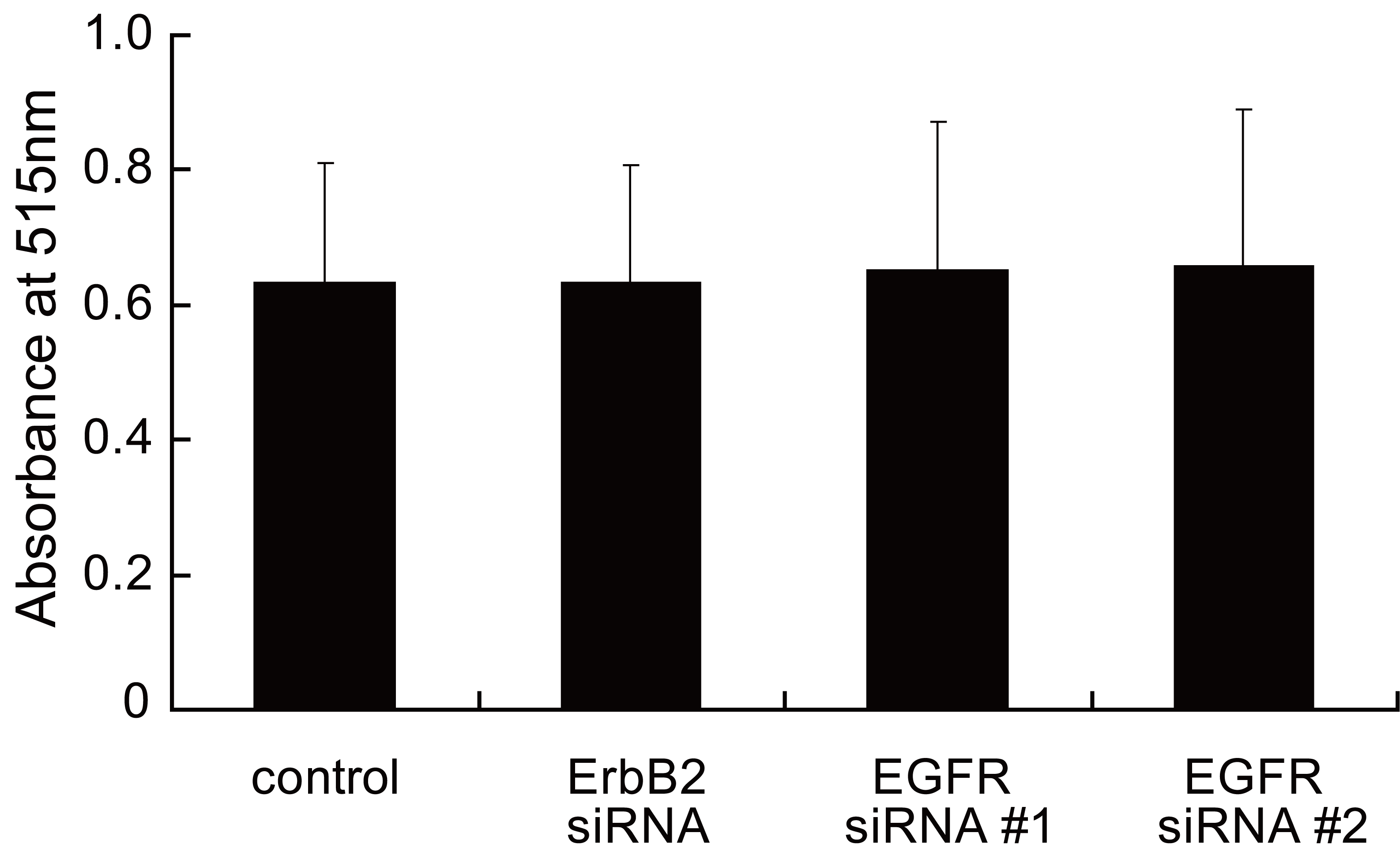

Supplement: Figure S2 — Different kinds of siRNA (50 nmol/L) were transfected to SK-OV-3 cells with oligofectamine reagent (Invitrogen) according to the manufacturer's instruction. Forty eight hours after transfection, growth effects of these siRNAs on the cells were determined by SRB assay. (TIF) [file pone.0021487.s002.tif]
