# Supplementary material for: AST1306, A Novel Irreversible Inhibitor of the Epidermal Growth Factor Receptor 1 and 2, Exhibits Antitumor Activity Both In Vitro and In Vivo
Source: PLoS One. 2011 Jul 18;6(7):e21487. doi: 10.1371/journal.pone.0021487 (PMC3138742; doi:10.1371/journal.pone.0021487)
Supplement: Text S1 — Detailed description of Materials and Methods. (DOC) [file pone.0021487.s003.doc]

**Materials and methods**

**Antibodies, kinases and reagents**

Antibodies against the following were used: phospho-EGFR and EGFR, phospho-ErbB2 and ErbB2, phospho-ERK1/2 and ERK1/2, phospho-AKT and AKT (all from Cell Signaling Technology, Beverly, MA, USA), GAPDH, actin and anti-phosphotyrosine (PY) antibody PY99 (Santa Cruz Biotechnology, Santa Cruz, CA, USA).

The kinase domain of EGFR, ErbB-2, IGF1R, VEGFR2, FGFR1, PDGFRα, c-Src, c-Met, RON, c-Kit, EphA2 and EphB2 were expressed using the Bac-to-Bac^TM^ baculovirus expression system (Invitrogen, Carlsbad, CA, USA) and purified on Ni-NTA columns (QIAGEN Inc., Valencia, CA, USA). VEGFR1, PDGFRβ and c-Abl were purchased from Upstate Biotechnology Inc. (Charlottesville, Virginia, USA). EGFR T790M/L858R was obtained from Cell Signaling Technology Inc, MA, U.S.A. Tie2, SYK, PKCb2, p70S6K, JAK2, Pim2, AKT1, AUR A, CDK2 and GSK3b were the products of Carna Biosciences, Inc (Chuo-ku, Japan). EGF was purchased from R&D, Minneapolis, Germany. Poly(Glu-Tyr)_(4:1)_, the substrate of tyrosine kinase, was from Sigma-Aldrich Corp. St. Louis, MO, USA. Lapatinib was purchased from Rong Da Pharma-chem Co., Ltd (Jiangsu, China).

**Compounds**

AST1306 was prepared as below: heating 2-chloro-4- nitropheno in DMF at 100 °C with 1-(bromomethyl)-3-fluorobenzene and potassium carbonate gave 3-chloro-4-(3-fluorobenzyloxy)-nitrobenzene which was reduced by refluxing with sodium sulfide in methanol to 3-chloro-4-(3-fluorobenzyloxy)-aniline. Chlorination of 6-nitro-quinazolin-4-one with oxalyl chloride in dichloromethane afforded 4-chloro-6-nitro-quinazoline. Reaction of 3-chloro-4-(3-fluorobenzyloxy) -aniline with 4-chloro-6-nitro-quinazoline in isopropyl alcohol obtained 4-[3-chloro- 4-(3-fluoro-benzyloxy)-phenylamino]-6- nitro-quinazoline, treatment of which with Pd/C in ethanol afforded 4-[3-chloro-4-(3-fluoro-benzyloxy) -phenylamino]-6-amino-quinazoline which was amidated by acrylic chloride in THF at room temperature gave N-{4-[3-chloro-4-(3-fluorobenzyloxy)-phenylamino]- quinazoling-6-yl}-acrylamide, treatment of which with p-toluenesulfonic acid afforded N-4-[3-chloro-4-(3-fluorobenzyloxy)-phenlylamino]-quinazolin-6-yl- acrylamide, 4-methylbenzenesulfonate (AST1306) (above 99% pure). ^1^H-NMR (DMSO-d6, 400MHz): *δ* 11.3 (s, 1H), 10.73 (s, 1H), 9.05 (s, 1H), 8.85 (s, 1H), 8.03-8.01 (dd, 1H), 7.84-7.81 (dd, 1H), 7.55-7.54 (d, 1H), 7.45-7.44 (dd, 1H), 7.32-7.27 (m, 1H), 7.16 (t, 1H), 7.08-7.06 (d, 2H), 6.51-6.47 (dd, 1H), 6.35-6.32 (d, 1H), 5.86-5.84 (d, 1H), 5.29 (s, 2H), 2.26 (s, 3H).

**Cell proliferation assay**

Cell proliferation was evaluated using the SRB (Sulforhodamine B) assay as previously described. Briefly, cells were seeded into 96-well plates and grown for 24 h. The cells were then treated with increasing concentrations of AST1306 and grown for a further 72 h. The medium remained unchanged until the completion of the experiment. The cells were then fixed with 10% precooled trichloroacetic acid (TCA) for 1 h at 4°C and stained for 15 min at room temperature with 100 μL of 4 mg/mL SRB solution (Sigma) in 1% acetic acid. The SRB was then removed, and the cells were quickly rinsed five times with 1% acetic acid. After cells were air-dried, protein-bound dye was dissolved in 150 μL of 10 mmol/L Tris base for 5 min and measured at 515 nm using a multiwell spectrophotometer (VERSAmax, Molecular Devices). The inhibition rate on cell proliferation was calculated as (1 - *A*_515 treated_/*A*_515 control_) × 100%. The IC_50_ value was obtained by the Logit method and was determined from the results of at least 3 independent tests.

**Tyrosine kinase assays**

The tyrosine kinase activities were determined in 96-well ELISA plates (Corning, NY, USA) precoated with 20 μg/mL Poly (Glu,Tyr)_4:1_. First, 80 μL of 5 μM ATP solution diluted in kinase reaction buffer (50 mM HEPES pH 7.4, 20 mM MgCl_2_, 0.1 mM MnCl_2_, 0.2 mM Na_3_VO_4_, 1 mM DTT) was added to each well. Various concentrations of AST1306 diluted in 10 μL of 1% DMSO (v/v) were then added to each reaction well, with 1% DMSO (v/v) used as the negative control. Subsequently, the kinase reaction was initiated by the addition of purified tyrosine kinase proteins diluted in 10 μL of kinase reaction buffer solution. Experiments at each concentration were performed in duplicate. After incubation for 60 min at 37 °C, the plate was washed three times with phosphate buffered saline (PBS) containing 0.1% Tween 20 (T-PBS). Next, 100 μL anti-phosphotyrosine antibody (PY99, 1:500 dilution) diluted in T-PBS containing 5 mg/mL BSA was added. After 30 min incubation at 37 °C, the plate was washed three times as before. Horseradish peroxidase-conjugated goat anti-mouse IgG (100 μL) diluted 1:2000 in T-PBS containing 5 mg/ml BSA was added. The plate was reincubated at 37 °C for 30 min, and then washed with PBS. Finally, 100 μL of a solution containing 0.03 % H_2_O_2_ and 2 mg/ml o-phenylenediamine in 0.1 M citrate buffer, pH 5.5, was added and samples were incubated at room temperature until color emerged. The reaction was terminated by the addition of 50 μL of 2 M H_2_SO_4_, and the plate was read using a multi-well spectrophotometer (MAX190™, Molecular Devices, Sunnyvale, USA) at 490 nm. The inhibition rate (%) was calculated using the following equation: [1-(A_490 treated_ /A_490 control_)] ×100%. IC_50_ values were determined from the results of at least three independent tests and calculated by Logit method.

**Western blot analysis**

For analysis of receptor tyrosine kinase phosphorylation and downstream signal transduction pathways in human cancer cells, cells were grown to half confluence in six-well plates and starved in serum-free medium for 24 h. AST1306 was added in serial dilutions, and the cells incubated for 4 h at 37 °C in medium without FBS. EGF (50 ng/mL, R&D, Minneapolis, Germany) was then added. After 15 min incubation at 37 °C, the cells were collected and suspended in lysis buffer (100 mmol/L Tris-HCl, pH 6.8, 200 mmol/L DTT, 4% SDS, 0.2% bromphenol blue, 20% glycerol). The cell lysate was cleared by centrifugation at 14,000 r/min for 15 min. Lysate proteins were resolved by sodium dodecylsulfate polyacrylamide gel electrophoresis and transferred onto nitrocellulose membranes. The membranes were incubated for 1 h in 5% milk, followed by 2 h of incubation with primary antibodies. The membranes were washed three times with PBS (with 0.1% Tween-20) and then incubated with the respective peroxidase-conjugated secondary antibody for 1 h. The membranes were washed again and developed using enhanced chemiluminescence (ECL, Amersham Biosciences), and then exposed to Kodak X-Omat BT film.

**Anchorage-independent cell transformation assay.** Cell transformation was detected in SK-OV-3 and A549 cells, respectively. Briefly, cells (8000 / mL) were treated with or without AST1306 in 1 mL of 0.3% basal medium Eagel agar (10% FBS). The culture were maintained in a 37°C, 5% CO2 incubator for 1-2 weeks, and the cell colonies were scored using a microscope and the Image-Pro PLUS computer software program (Media Cybernetics, Silver Spring, MD). Data are shown as means ± SD of values from triplicate experiments and the asterisk (*) indicates a significant (**P*<0.05, ** *P*<0.01) decrease in colony number compared to untreated control.
